# Supplementary material for: Multifunctional Biomass-Based Ionic Liquids/CuCl-Catalyzed CO2-Promoted Hydration of Propargylic Alcohols: A Green Synthesis of α-Hydroxy Ketones
Source: Int J Mol Sci. 2024 Feb 5;25(3):1937. doi: 10.3390/ijms25031937 (PMC10856482; doi:10.3390/ijms25031937)
Supplement: Supplementary file 1 [file ijms-25-01937-s001.zip › ijms-2830919-supplementary.pdf]

Supplementary Files

for

Multifunctional Biomass-based Ionic Liquids/CuCl Catalyzed CO_2-promoted Hydration of Propargylic Alcohols: A Green Synthesis of α-Hydroxy Ketones

Ye Yuan,[a, b] Siqi Zhang,[b] Kang Duan,[b] Yong Xu,[b] Kaixuan Guo,[b] Cheng Chen,[a, b] Somboon Chaemchuen,[a, b] Dongfeng Cao,[a, b, *] Francis Verpoort,[a, b, c,*]

[a] State Key Laboratory of Advanced Technology for Materials Synthesis and Processing, Wuhan University of Technology, Wuhan 430070, PR China

[b] School of Material Science and Engineering, Wuhan University of Technology, Wuhan 430070, PR China

[c] National Research Tomsk Polytechnic University, Lenin Avenue 30, 634050 Tomsk, Russia

Corresponding Authors: Dongfeng Cao (D. Cao): cao_dongf@whut.edu.cn; Francis Verpoort (F. Verpoort): francis@whut.edu.cn

1. Synthesis of the imidazole-based ILs

The imidazole-based ILs was synthesized based on the ion exchange resin methods, as reported in literatures with modifications. [1-2] Taking [C_2C_1im][Lev] as an example, 20 mmol [C_2C_1im][Br] and 45 mL anhydrous ethanol were added in a 100 mL round-bottomed flask. Upon complete dissolution, 20 mL of anion-exchange resin (D201 OH, supplied by Ningbo Zhengguang resin Co., LTD) was added and the mixture was stirred at room temperature for 6 hours. Subsequently, the resin was separated and washed 3-5 times with total 20 mL anhydrous ethanol in a Bush funnel. The obtained [C_2C_1im][OH] filtrate was concentrated to 50 mL and added with 20 mmol levulinic acid. The mixture continued to stir at room temperature for 12 hours, then the solvents in the mixture were totally removed under vacuum to obtain the final [C_2C_1im][Lev].

2. Calculation of green metrics

“**1a**” in Table 3 (entry 1) was selected as examples to show how AE, E-factor, CE, RME, MI and MP were calculated:

The reaction of **1a** (2.5 mmol, 0.2103 g), H_2O (5 mmol, 0.09 g) and equivalent CO_2 (2.5 mmol, 0.044 g) were carried out with the employment of 1 mol% of CuCl (0.025 mmol, 0.00247 g) and 1 equiv. of [C_2C_1im][Lev] (2.5 mmol, 0.5682 g). **2a** (95%) was obtained.

(1)

$$AE\ (\%) = \frac{Mole\ weight\ of\ product \times 100}{Sum\ of\ mole\ weights\ of\ reactants}$$

$$= \frac{102.13 \times 100}{84.12 + 18.02}$$

$$= 100.0$$

(2)

$$MI = \frac{Total\ mass\ in\ process}{Mass\ of\ product}$$

$$= \frac{0.2103 + 0.09 + 0.002474 + 0.5682 + 0.11}{0.0025 \times 0.95 \times 102.13}$$

$$= 4.04$$

(3)

$$CE\ (\%) = \frac{Carbon\ in\ product \times 100}{Total\ carbon\ in\ reactants}$$

$$= \frac{5 \times 0.95 \times 2.5}{2.5 \times 5} \times 100$$

$$= 95$$

(4)

$$E\ = \frac{Total\ mass\ of\ waste}{Mass\ of\ final\ product}$$

$$= MI - 1$$

$$= 3.04$$

(5)

$$ME\ (\%) = \frac{Mass\ of\ product \times 100}{Total\ mass\ of\ reactants}$$

$$= \frac{0.0025 \times 0.95 \times 102.13}{0.2103 + 0.09} \times 100$$

$$= 80.8$$

(6)

$$MP\ (\%) = \frac{Mass\ of\ product \times 100}{Total\ mass}$$

$$= \frac{1}{MI} \times 100$$

$$= \frac{1}{4.04} \times 100$$

$$= 24.7$$

3.NMR spectra of the products and ionic liquids

^{1}H NMR of **2a**

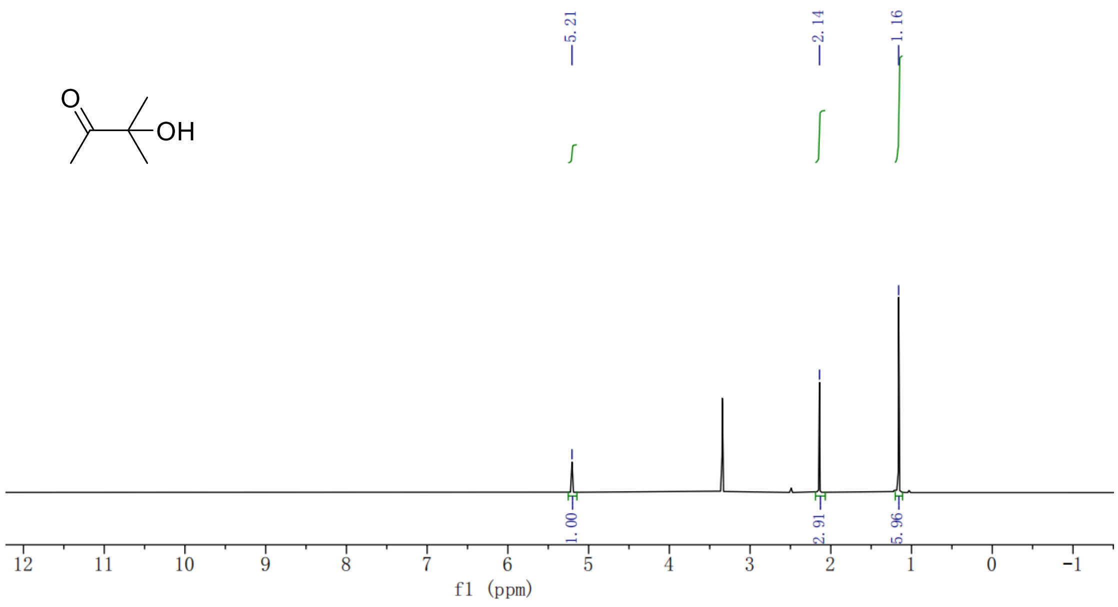

^{13}C NMR of **2a**

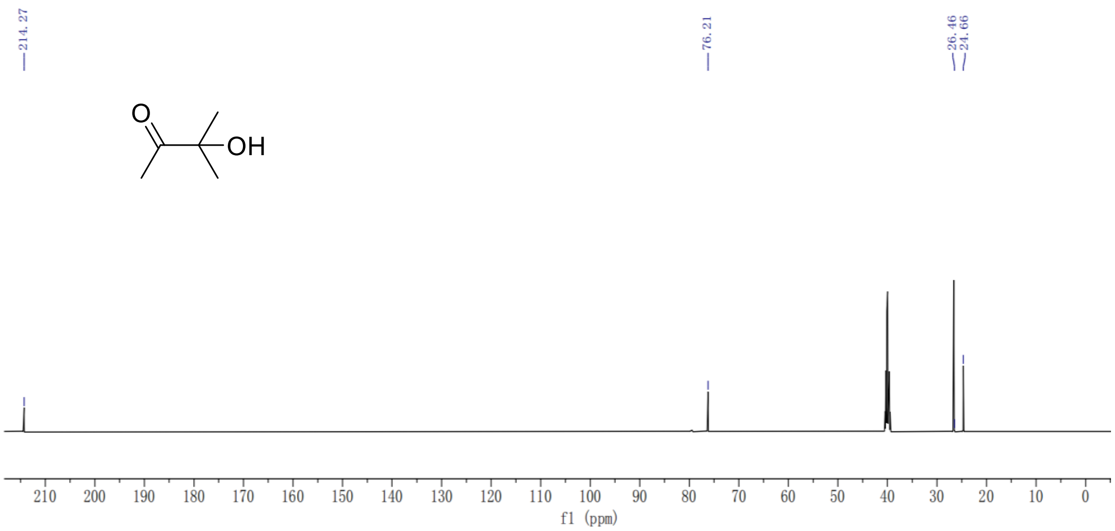

These spectra are matched with the reported publication [3].

^{1}H NMR of **2b**

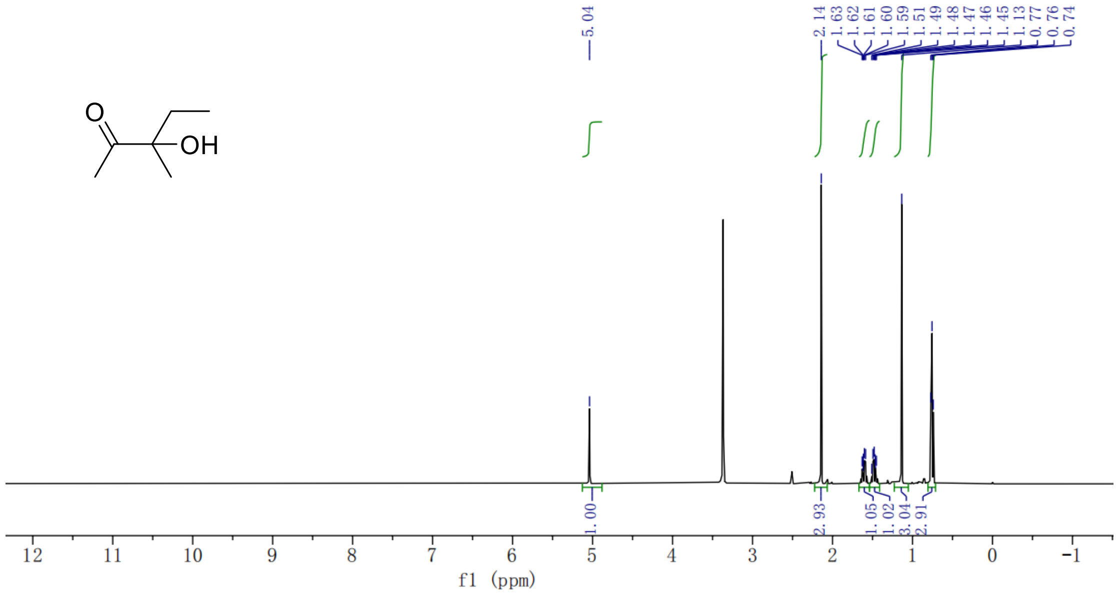

^{13}C NMR of **2b**

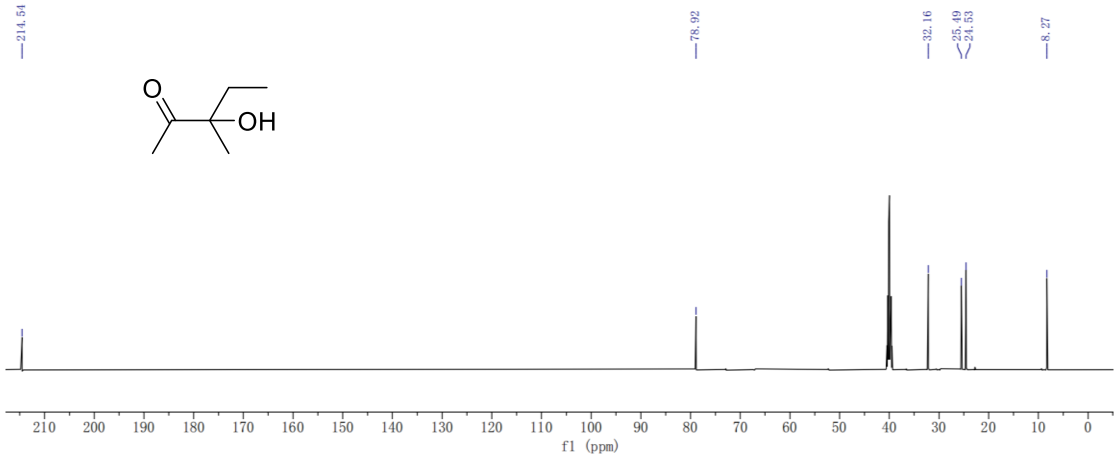

These spectra are matched with the reported publication [3].

^{1}H NMR of **2c**

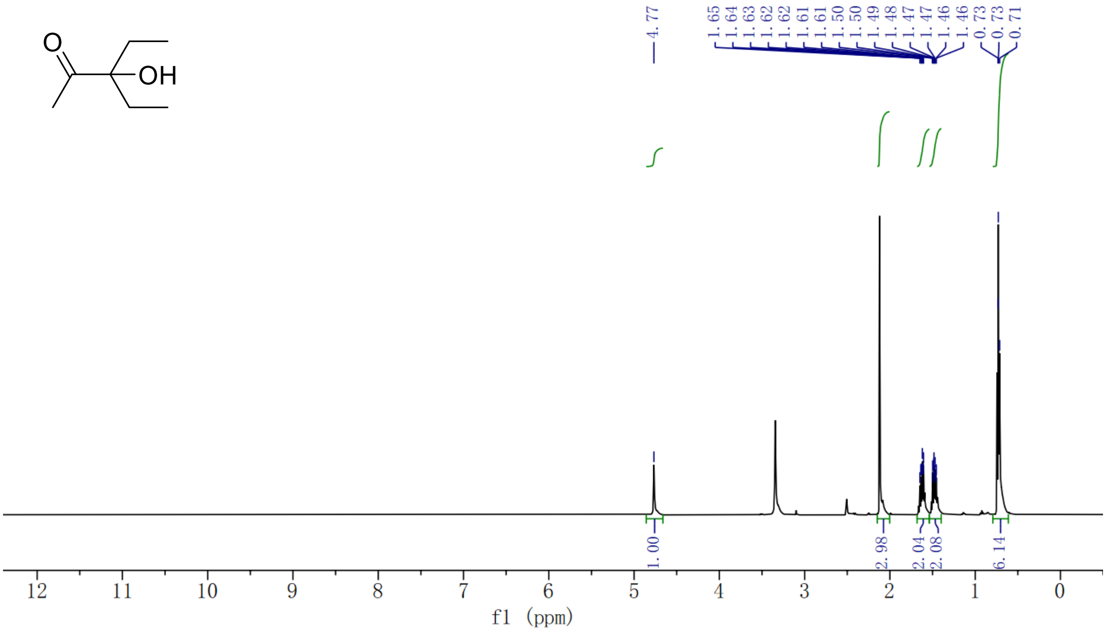

^{13}C NMR of **2c**

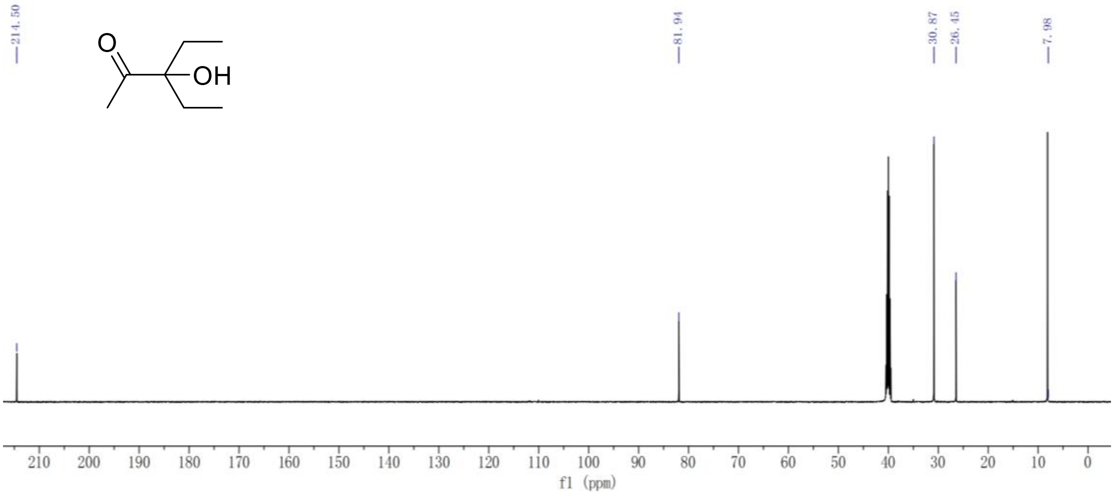

These spectra are matched with the reported publication [3]

^{1}H NMR of **2d**

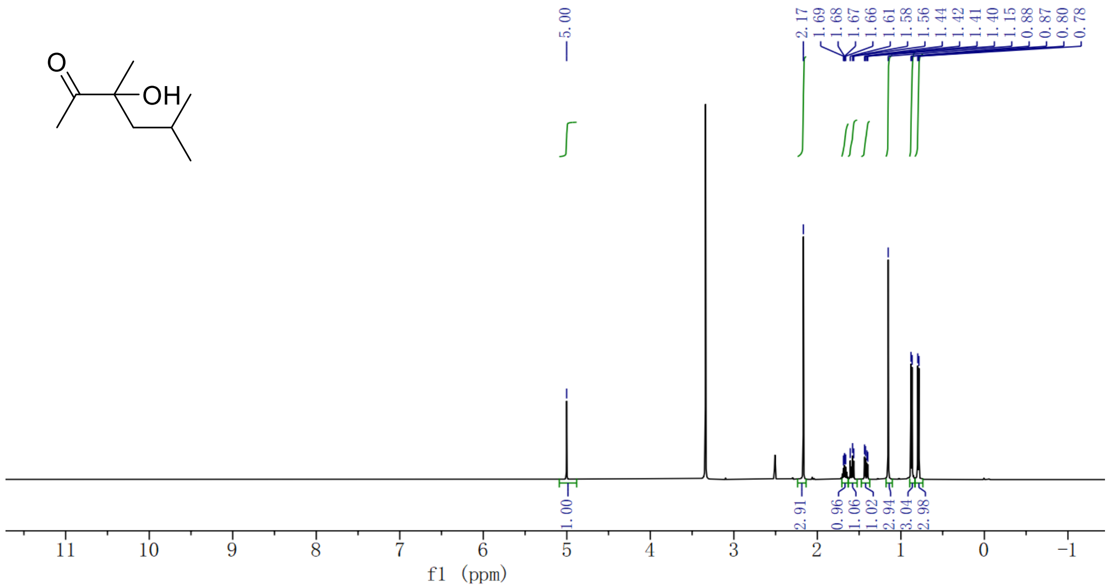

^{13}C NMR of **2d**

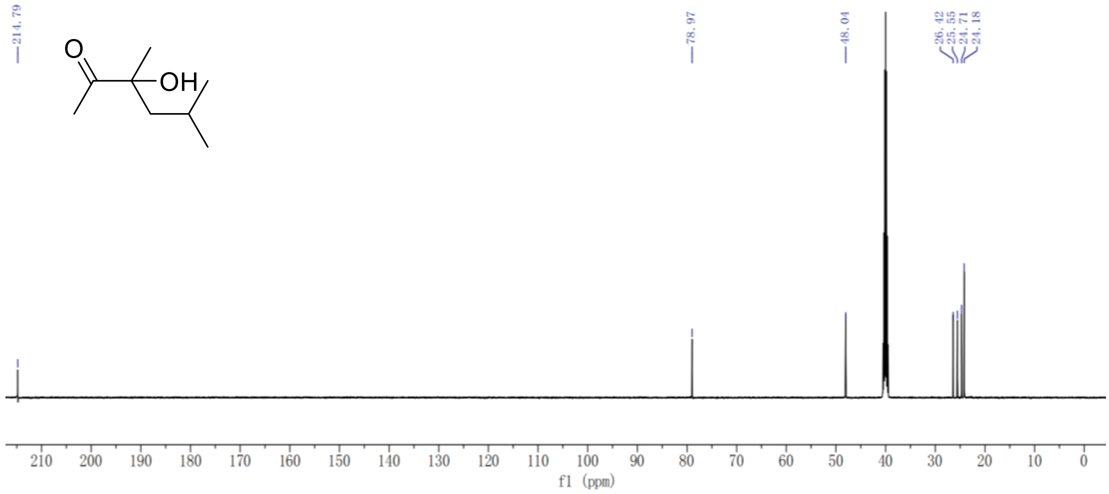

These spectra are matched with the reported publication [3]

^{1}H NMR of **2e**

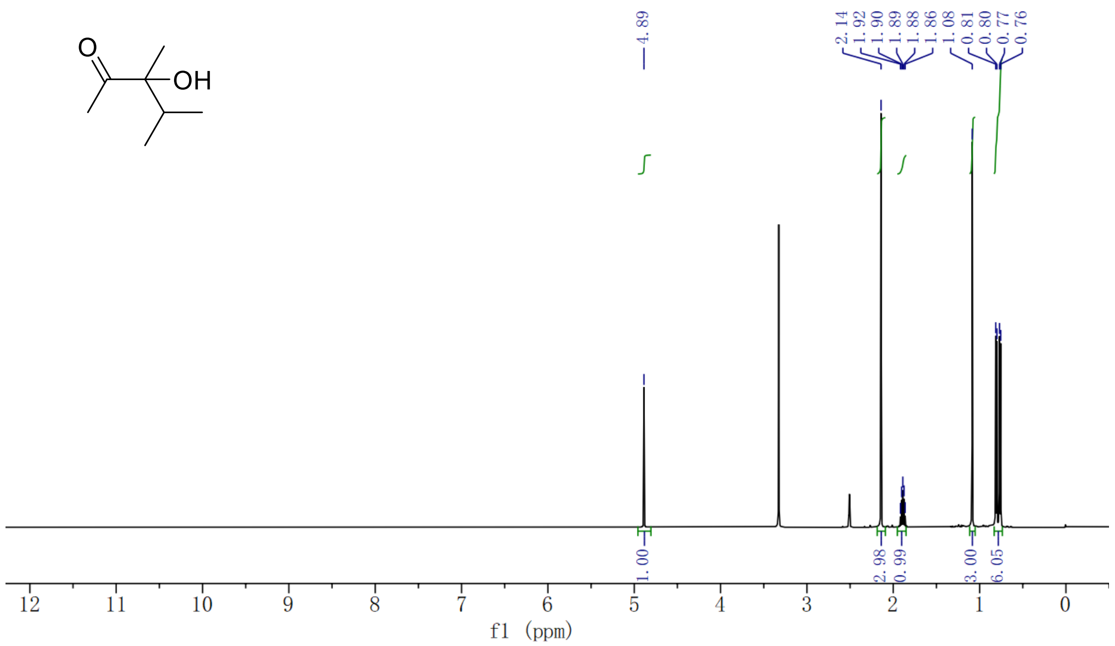

^{13}C NMR of **2e**

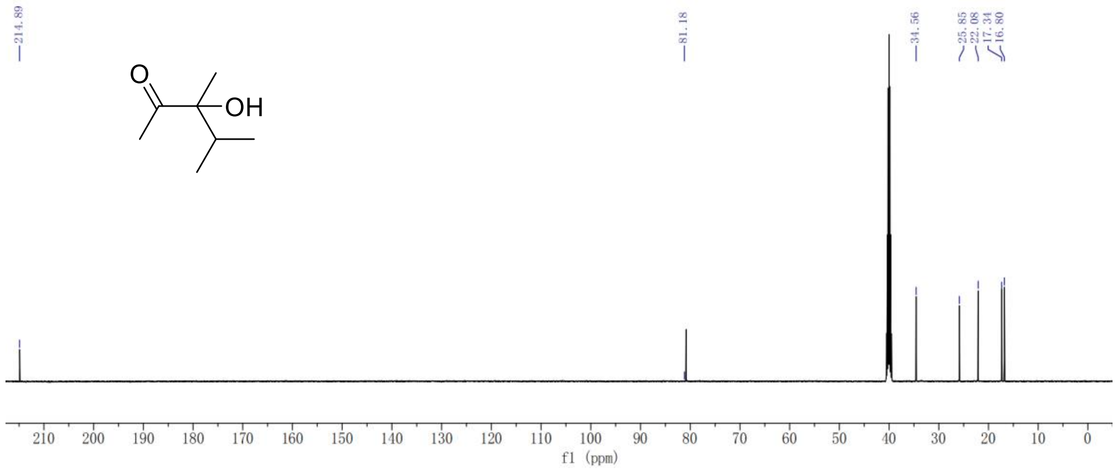

^{1}H NMR of **2f**

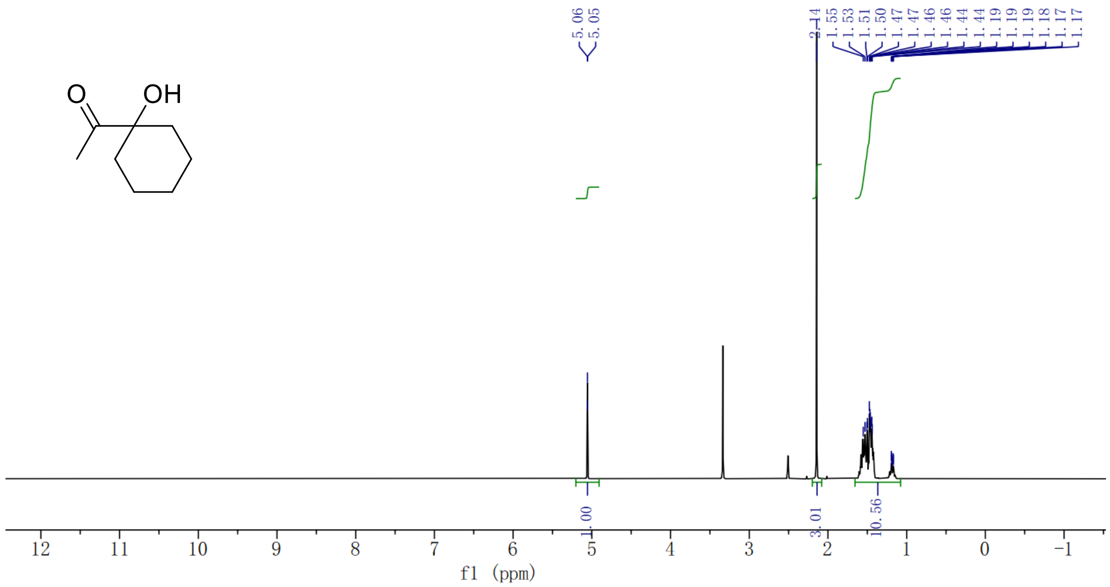

^{13}C NMR of **2f**

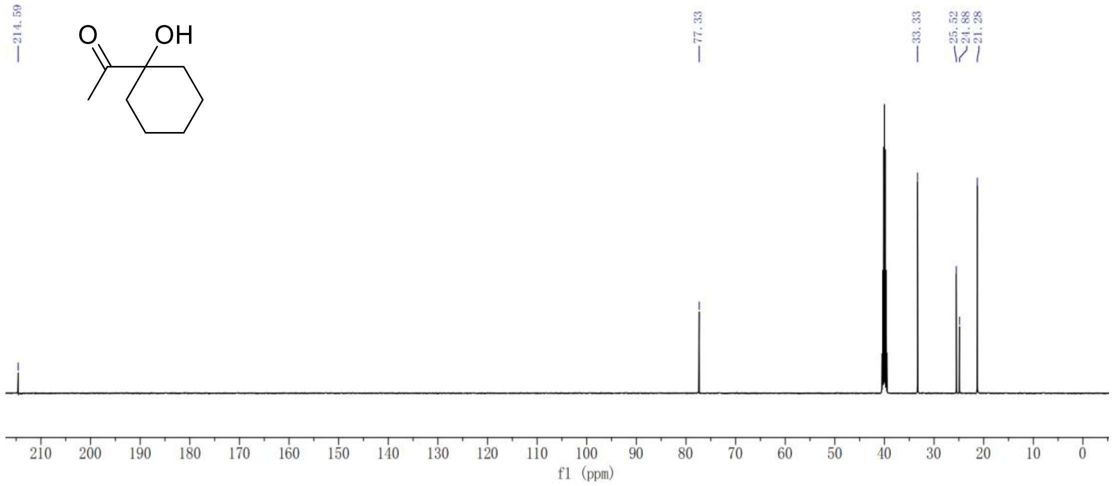

These spectra are matched with the reported publication [3]

^{1}H NMR of **2g**

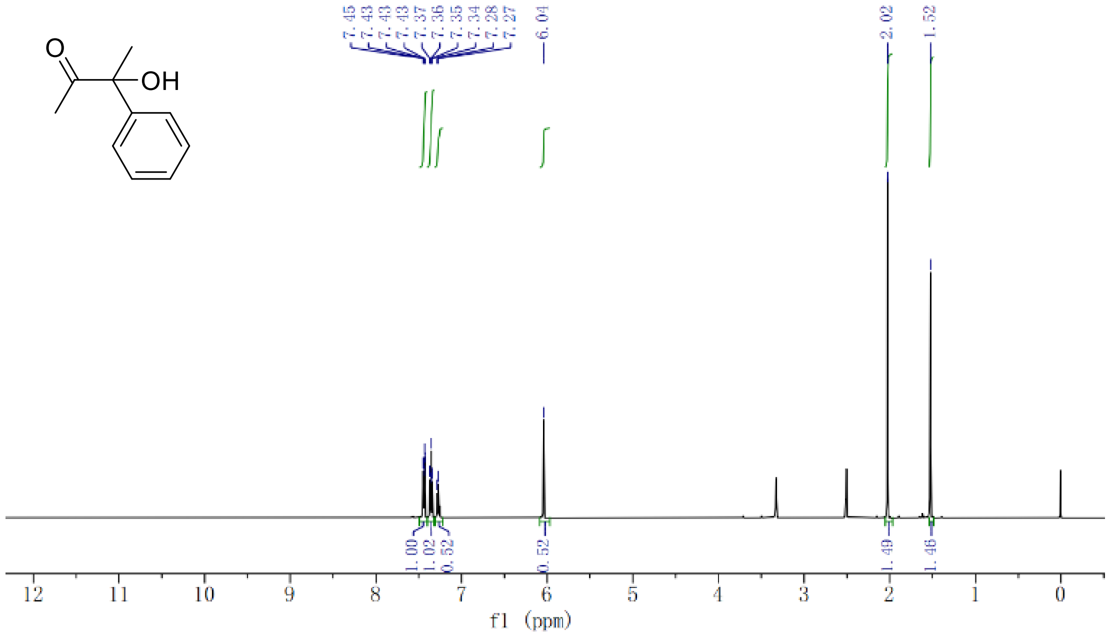

^{13}C NMR of **2g**

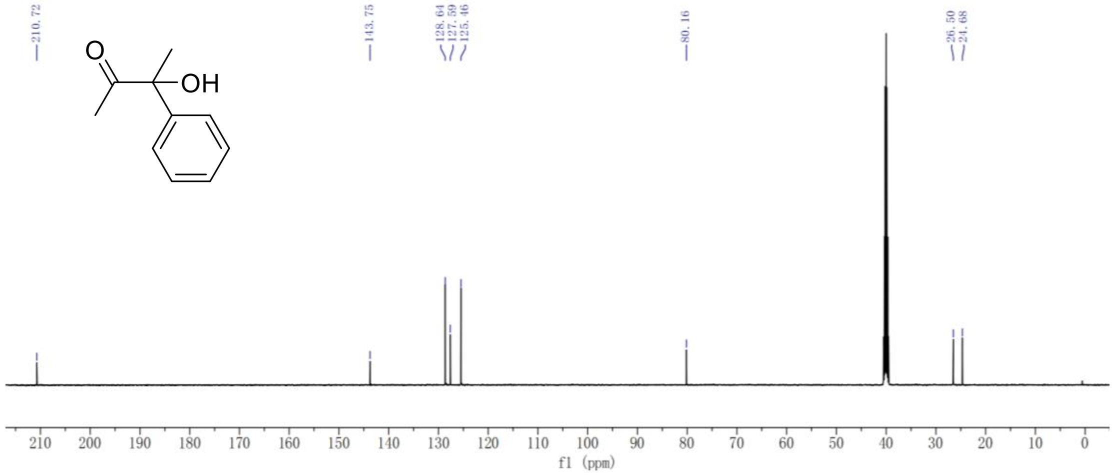

These spectra are matched with the reported publication [3]

^{1}H NMR of [C_2C_1im][Lev]

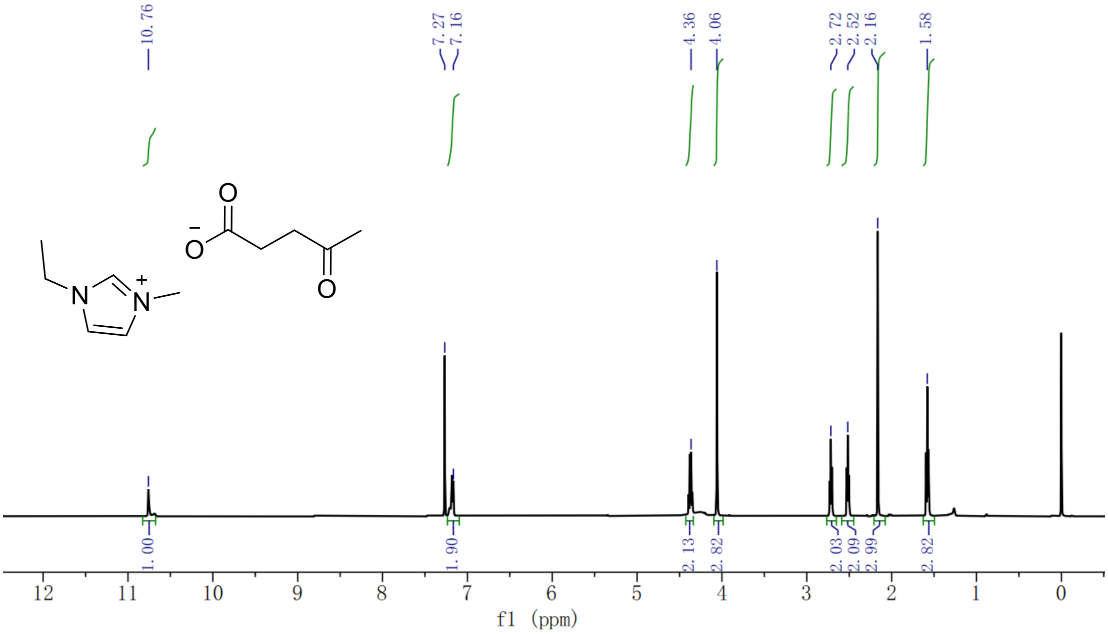

^{13}C NMR of [C_2C_1im][Lev]

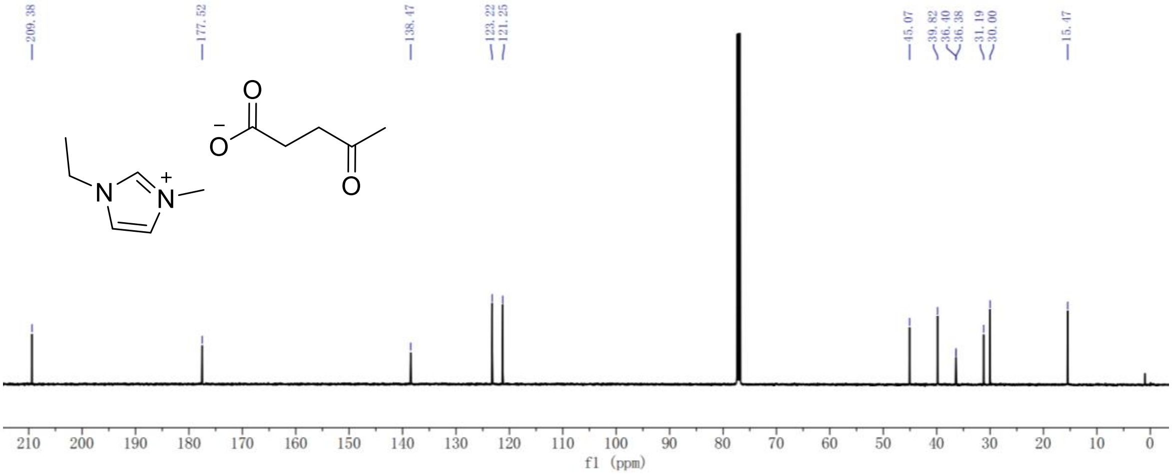

4. References

1. Hu, Y.; Song, J.; Xie, C.; Wu, H.; Jiang, T.; Yang, G.; Han, B., Transformation of CO_2 into α-Alkylidene Cyclic Carbonates at Room Temperature Cocatalyzed by CuI and Ionic Liquid with Biomass-Derived Levulinate Anion. *ACS Sustainable Chem. Eng.* **2019,** *7* (6), 5614-5619.
2. Mezzetta, A.; Becherini, S.; Pretti, C.; Monni, G.; Casu, V.; Chiappe, C.; Guazzelli, L., Insights into the levulinate-based ionic liquid class: synthesis, cellulose dissolution evaluation and ecotoxicity assessment. *New J. Chem.* **2019,** *43* (33), 13010-13019.
3. Zhao, Y.; Yang, Z.; Yu, B.; Zhang, H.; Xu, H.; Hao, L.; Han, B.; Liu, Z., Task-specific ionic liquid and CO_2-cocatalysed efficient hydration of propargylic alcohols to α-hydroxy ketones. *Chem. Sci.* **2015,** *6* (4), 2297-2301.
